# Long-term health of children conceived after assisted reproductive technology

**DOI:** 10.1080/03009734.2020.1729904

**Published:** 2020-02-26

**Authors:** Christina Bergh, Ulla-Britt Wennerholm

**Affiliations:** a Department of Obstetrics and Gynecology, Institute of Clinical Sciences, Sahlgrenska Academy, Gothenburg University, Gothenburg, Sweden;; b Reproductive Medicine, Sahlgrenska University Hospital, Gothenburg, Sweden

**Keywords:** Assisted reproductive technology, long-term follow up, ICSI, IVF

## Abstract

The aim of this narrative review is to summarize the present knowledge on long-term outcome of children born after assisted reproductive technologies (ART). The main outcomes covered are neurodevelopment including cerebral palsy, cognitive development, attention deficit hyperactivity disorder and autism spectrum disease, growth, cardiovascular function, diabetes type 1, asthma, malignancies, and reproductive health. Results have mainly been obtained from systematic reviews/meta-analyses and large registry studies. It has been shown that children born after ART, when restricted to singletons, have a similar outcome for many health conditions as their spontaneously conceived peers. For some outcomes, particularly cardiovascular function and diabetes, studies show some higher risk for ART singletons or subgroup of ART singletons. The fast introduction of new ART techniques emphasizes the importance of continuous surveillance of children born after ART.

## Introduction

There are numerous publications on the short-term outcome after assisted reproductive technology (ART), including large registry studies and systematic reviews (SRs)/meta-analyses. Most of them reveal some adverse outcome also for singletons, particularly concerning preterm birth, low birth weight, and birth defects. Literature concerning long-term outcome is much more sparse. This is the case, despite the fact that large cohorts of ART children and adolescents now exist. While national birth registries, with extensive data on neonatal outcome, are present in several countries, this is not the case for children’s health in general. To catch children/adolescence health, patient or specific disease registries may be used. These registries include, however, only those children with specific diseases, and most severe diseases occur later in life, leaving few events and low power of such studies. The following summary is based on large registry studies and SRs where such exist. Cohort studies with sometimes a limited number of children have been included as well. The following aspects will be discussed: neurodevelopment including cerebral palsy (CP), cognitive development, attention deficit hyperactivity disorder (ADHD) and autism spectrum disease (ASD), growth, cardiovascular function, diabetes type 1, asthma, malignancies, and reproductive health.

## Neurodevelopmental health

### Psychomotor and language development, behaviour, and social functioning

Three SRs from the Netherlands, Denmark, and Australia described no differences in psychomotor development, overall social functioning, language development, and behaviour between children born after ART and spontaneously conceived controls (1–3).

### Cognitive development

A recent SR focussing on cognitive development following ART (4) found conflicting results, mainly due to methodological limitations. However, three studies were considered high-quality studies and suggested ART to have some negative influence on cognitive development (5–7). In the early study by Strömberg and co-workers (5) including 5,680 children born after IVF (both singletons and multiples), a 4-fold increase in risk of developmental delay was observed as well as an increased risk of needing habilitation services compared with children from spontaneous conception. When comparing only singletons, there was no increased risk. In a Dutch study with a limited number of ART children, singletons from intracytoplasmic sperm injection (ICSI) were shown to have lower scores on tests of intelligence (on average 5–7 IQ points lower) compared with spontaneously conceived singletons (6). In a large population-based registry study (*n* = 30,959 ART children) from Sweden a small but significantly increased risk of mental retardation was found in ART children (relative risk [RR] 1.18, 95% CI 1.01–1.36) (7). When restricting analysis to singletons statistical significance disappeared. Further, in a subgroup analysis of singletons after frozen/thawed ICSI, there was an increased risk of mental retardation; however, this was based on few children (*n* = 7). A Danish registry study reported comparable risk of mental retardation in IVF-conceived and spontaneously conceived singletons (8).

Recently, large registry-based studies from Sweden and Denmark have shown similar school performances of ART children and children born from spontaneous conception. The Swedish study (9) included 8,323 ART singletons compared with 1,499,667 children born after spontaneous conception, representing all ART and spontaneously conceived children born in Sweden between 1985 and 2001. Interestingly, the mean scores were around 10% higher for the ART children. However, after adjustment for relevant confounders, results were similar in the two groups. Comparisons have also been made between children from IVF and ICSI and between children from fresh and frozen cycles (10–12), with similar results.

### Attention deficit hyperactivity disorder and autism spectrum disease

ADHD was found to be weakly associated with IVF in a Swedish study including 28,158 multiples and singletons born after IVF. Yet after adjusting for length of involuntary childlessness, or when only singletons were analyzed, the statistical significance disappeared (13). A Danish study including 124,269 children born to women with fertility problems reported a higher risk of ADHD (hazard ratio [HR] 1.36, 95% CI 1.29–1.45) in these children compared with children born to women without fertility problems. No adjustment was made for multiple gestations in that study (14).

In a Swedish study, including 30,959 children born after ART, no increased risk of ASD was found compared to children born after spontaneous conception (7). Nor was there an increase in any emotional and behavioural disorder in a large Danish study (8). However, in a Californian cohort study a higher risk of ASD was reported in ICSI singletons compared with standard IVF with fresh embryo transfer (ET) (adjusted hazard risk ratio [aHR] 1.65, 95% CI 1.08–2.52) (ART *n* = 19,790) (15). No comparison between children from ART and spontaneous conception was performed in that study (15). In a recent meta-analysis it was concluded that ART was associated with a greater risk of ASD in an overall offspring group compared with spontaneously conceived children (RR 1.35, 95% CI 1.09–1.68); however, this was not seen in singletons (16).

### Cerebral palsy (CP)

An increased risk of CP was found for singletons in an early Swedish study including 5,680 children born after IVF (aOR 2.8, 95% CI 1.3–5.8) (5). In a later and larger Swedish study, including 31,614 children born after IVF (multiples and singletons), born 1982–2007, there was a higher risk for CP when analysing singletons and multiple birth children together and compared with all children born during the same time period and registered in the Medical Birth Registry (aOR 1.81, 95% CI 1.52–2.13). The analysis was based on 138 ART children with CP, and adjustments were made for maternal age, year of birth, parity, and smoking. When only singletons were analysed, no significant increased risk was found (17). Likewise, in a Danish study there was an increased risk for CP among 33,139 IVF children, but this disappeared when adjusted for multiplicity and gestational age (18). Another registry-based Danish study, however, including 10,329 singletons after fresh cycles and a control group of 4,800 spontaneously conceived singletons randomly selected, proved an increased risk of CP in singletons after fresh transfer (based on 42 CP cases) compared with spontaneously conceived singletons (aOR 2.44, 95% CI 1.15–5.22) (19). However, no adjustment was made for gestational age. In a recent Australian register study including a limited number of children (*n* = 2,914) born after ART, the prevalence of CP was more than doubled in ART singletons born very preterm (<32 gestational weeks) (20).

*In summary*, for most neurodevelopmental health variables conflicting results exist concerning a possible association between ART and adverse outcome. Most of the identified risk associations disappeared after adjustment for multiple births or were observed only in subgroups of specific IVF treatments such as cryopreservation. For CP, in particular, there is a need of further large studies, including more recent cohorts of ART children.

## Cardiovascular function and metabolism

The literature on cardiovascular and metabolic risks in ART offspring is limited. Studies published so far are based on small cohorts with high risk for selection bias among both ART children and controls. Cardiovascular and metabolic diseases mainly affect adults, while adolescents and young adults conceived after ART are not very common in registries covering these diseases.

The most recent SR and meta-analysis on cardiovascular diseases included 19 studies with 2,112 IVF/ICSI and 4,096 spontaneously conceived offspring (21). It was found that systolic blood pressure (SBP) and diastolic BP (DBP) levels were higher after ART than in spontaneously conceived offspring (weighted mean differences [WMD] 1.88, 95% CI 0.27–3.49 for SBP; and 1.51, 95% CI 0.33–2.70 for DBP). However, the higher WMD in SBP and DBP was only observed in the cohorts born 1990–1999 and not for children born later.

The same meta-analysis showed comparable body mass index (BMI), low-density lipoprotein, cholesterol, and fasting insulin levels for ART and spontaneously conceived children.

A Swiss study examined 65 ART singletons and 57 spontaneously conceived controls at 11–12 years of age and demonstrated adverse vascular dysfunction among ART children (22). These differences still existed at re-examination 5 years later when the children were 16–17 years old. Further, the ART children had significantly higher SBP and DBP (23).

*In summary*, limited data suggest a potential increase in blood pressure in ART children as well as suboptimal cardiovascular function.

### Diabetes type 1

Few studies have been published concerning the risk of developing diabetes type 1 in children conceived by ART. According to a Danish cohort study from 2016, including 8,490 IVF and ICSI children, there was no increased risk of diabetes type 1 (24). In a recent large registry study from Sweden, including 47,938 ART singletons and 3,090,602 singletons from spontaneous conception, there were 202 children born after ART that developed diabetes type 1 (25). The corresponding figure for children born after spontaneous conception was 17,916. This corresponds to 43.4 (ART) and 35.5 (spontaneous conception) children when calculated per 100,000 person-years. After adjustment for several confounders, including maternal and paternal diabetes, there was no overall difference between ART and spontaneous conception (aHR 1.07, 95% CI 0.93–1.23). However, children conceived after frozen/thawed transfers had a higher risk of developing diabetes type 1, both compared with singletons from fresh transfers and children from spontaneous conceptions (aHR 1.52, 95% CI 1.08–2.14; and aHR 1.41, 95% CI 1.05–1.89).

*In summary*, information on the occurrence of diabetes type 1 in ART children is scarce. In general, there are no alarming results for ART children, although there might be an increased risk for children born after frozen/thawed cycles.

## Growth

Though children born after ART are more likely to be born preterm and with low birth weight, catch-up is common during the first year of life, and most studies have shown similar growth patterns in ART and children spontaneously conceived. In a recent SR and meta-analysis from Denmark (26), weight and height of ART singletons compared with spontaneously conceived singletons were summarized. The SR included 20 studies, altogether 3,972 ART children and 11,012 spontaneously conceived children, followed up for a maximum of 22 years. There were no significant differences in weight or height.

*In summary*, so far, overall results on growth in children born after ART suggest that they do not differ from spontaneously conceived children.

## Respiratory disorders

Few studies have investigated the risk of asthma in ART children. A Swedish registry study of 2,628,728 children born in 1982–2007 including 31,918 children conceived by ART revealed an increased risk for asthma in children born after ART, increasing the absolute risk from 4.4% to 5.6% (27). However, adjustment for the duration of infertility eliminated the effect, suggesting that the main risk factor seemed to be subfertility, included in the characteristics of women in ART. A UK prospective study, the Millennium Cohort Study, of 18,818 singletons born after spontaneous conception or different kinds of fertility treatment, recruited at 9 months of age and based on a follow-up survey at 5 and 7 years of age, found a significant association between ART and asthma; however, this was based on few children after ART (28).

*In summary*, limited data suggest that the main risk factor for the association between asthma and ART is parental subfertility, but neonatal morbidity and maternal asthma may act as mediators.

## Malignancies

A population-based British cohort study from 2013, including 106,013 ART children followed for a mean of 6.6 years, proved no increase in the overall risk of cancer when compared with the expected risk for singleton births (standardized incidence ratio [SIR] 0.98, 95% CI 0.81–1.19) (29). Another large registry-based cohort study, combining data from four Nordic countries (CoNARTaS, Committee of Nordic ART and Safety) including 91,796 children (singletons and multiples) born after ART and 358,419 children born after spontaneous conception, also concluded that there was no increase in overall cancer rates among ART children (aHR 1.08, 95% CI 0.91–1.27) (30). Children in this cohort were born 1982–2007 and were followed for a mean of 9.5 years. In a large cohort study from the United States, published in 2019, 275,686 children born after ART and 2,266,847 children born after spontaneous conception were followed for 4.5 and 4.7 years, respectively (31). Per 1,000,000 person-years the incidence of cancer among ART was 251.9 cases and among spontaneously conceived children 192.7 cases. In total 321 and 2,042 children with cancer were identified among ART and spontaneously conceived children, respectively (aHR 1.17, 95% CI 1.00–1.36). While no overall significant increase in cancer was observed, there was an increase of hepatic cancer and embryonal tumours among ART children in two sub-analyses. A recent study from the Netherlands, including 24,268 ART children and 13,761 children from spontaneous conceptions, but also including a control group of 9,660 children from subfertile couples, did not observe any increase in risk of cancer among the ART children (32). The study period was between 1980 and 2001, and the children were followed for a mean of 21 years. More recently, a study from Denmark, including 1,085,172 children born between 1996 and 2012 and followed up to 2015, investigated a possible association between use of fertility drugs, assisted reproduction, and cancer in childhood (33). The study included 19,448 IVF, 13,427 ICSI, and 3,356 children from cryopreservation, and a control group of 910,291 children born to fertile women. The mean follow-up was 11.3 years. No overall risk of cancer was found among children born to women with any fertility problems, any fertility treatment, use of any fertility drugs, or use of any ART. However, an increased risk was observed among children born after cryopreservation of embryos (aHR 2.43, 95% CI 1.44–4.1). This observation was, however, based on only 14 cases.

*In summary*, there is some inconsistency concerning malignancies in children conceived by ART. Higher risks have mainly been observed in subgroups of patients and for specific types of malignancies.

## Reproductive health

Researchers from Brussels have published the first studies on the reproductive outcome in ART offspring. In one study among 54 young adult men conceived by ICSI because of severe male infertility, it was found that sperm parameters were significantly lower in ART men than in men after spontaneous conception. There was, however, no clear correlation between semen parameters of the young ICSI men and their fathers (34). The same authors showed similar levels of reproductive hormones among ART and spontaneously conceived men, and also similar levels of reproductive hormones, antral follicle count, and levels of anti-Müllerian hormone in women conceived after ICSI (35,36).

*In summary*, limited data published on reproductive health in ART offspring suggest some deterioration in sperm counts in ICSI male offspring, while in female offspring no adverse effects have been identified.

## Comments and conclusions

Follow-up studies of children born after ART have shown that the majority of children are healthy, even though some adverse outcomes have been demonstrated (Table 1). The main risk for adverse outcomes in ART, which includes ICSI and standard IVF techniques as well as cryopreservation techniques, has been associated with the higher rates of multiple pregnancies in ART. Sweden has been the pioneering country in the world concerning introduction of single embryo transfer as the main strategy to increase the health of children born after ART (see www.qivf.se) (37).

**Table 1.** Summary of associations between ART and children’s health and morbidity (versus spontaneous conception).

	ART		IVF	ICSI	Cryopreservation
Disorder	general	singletons			
Psychomotor and language development, behaviour, and social functioning	No association	No association			
Cognitive development		No association	No association	No association	No association
ADHD and ASD	Increased risk	No association			
Cerebral palsy	Increased risk	Conflicting results			
Cardiovascular function, blood pressure		Increased risk			
Diabetes type 1		No association	No association	No association	Increased risk
Growth		No association			
Asthma	No association				
Childhood malignancies	Conflicting results	No association			Increased risk
Reproductive health				Some sperm problems in men, no problems in women	

Concerning long-term effects of ART on children outcomes, few studies of high quality exist. Studies of growth seem reassuring. For childhood cancer, some discrepancies exist, but most large studies do not show any increase in childhood cancer in ART children after adjustment for relevant confounders. Most studies on neurocognitive development and ASD show no increased risks if adjusted for multiple births. School performances of 15–16-year-old adolescents have been investigated in large registry studies from Denmark and Sweden and have shown better school performance for ART children in crude analyses, but after adjustment for relevant confounders, particularly parental education, no differences of clinical importance have been observed. There are some recent concerns regarding cardiovascular parameters. Cohort studies with a limited number of children included have detected altered blood vessel structure and increased blood pressure, both SBP and DBP, in ART singletons compared with matched controls and further that these differences remain in adolescence. For diabetes type 1, in a recent Swedish large registry study, there was no increase in general for ART children but a higher risk in children born after the use of cryopreserved embryos.

An important bias in all these follow-up studies is the choice of control group, being children from the general population in most studies. It is known that infertility *per se* is associated with an adverse child outcome, which may well contribute to the findings of a poorer outcome among ART children. Control groups with subfertile couples are, however, difficult to find. Sibling studies (38,39), where the mothers have given birth to both an ART and a spontaneously conceived child suggest that both the infertility and the ART technique as such contribute to the child outcome.

In conclusion, even though many studies on follow-up of ART children show reassuring results, information on long-term follow-up is limited. New and advanced techniques are rapidly introduced and implemented, emphasising the importance of continuous surveillance of children born after ART.
